# The minimum effective concentration (MEC90) of ropivacaine for ultrasound-guided quadratus lumborum block for analgesia after cesarean delivery: a dose finding study

**DOI:** 10.1186/s12871-022-01954-5

**Published:** 2022-12-29

**Authors:** Rong Cao, Xuehan Li, Jing Yang, Lingmei Deng, Yu Cui

**Affiliations:** 1 Department of Anesthesiology, The Affiliated Hospital, School of Medicine, UESTC Chengdu Women’s and Children’s Central Hospital, Chengdu, 610091 China; 2 Department of Anesthesiology, and Laboratory of Anesthesia and Intensive Care Medicine, West China Hospital of Sichuan University, Chengdu, China

**Keywords:** Quadratus lumborum block, Minimum effective concentration, Cesarean delivery, Postoperative pain

## Abstract

**Background:**

Quadratus lumborum block was recently proposed as an alternative technique for post-cesarean delivery analgesia. However, there is not a definite optimum concentration of local anesthetics. A biased coin design up-and-down method was used to explore the minimum effective concentration of ropivacaine in quadratus lumborum block for satisfactory analgesia after cesarean delivery.

**Methods:**

Fifty-six patients weighing 60–80 kg after cesarean section and with ages between 18 and 40 years were recruited. For the posterior quadratus lumborum block, a volume of 25 ml of the assigned concentration of ropivacaine was injected bilaterally.

The concentration administered to each patient depended on the response to the previous dose. The first patient received 0.25%. If a successful block was observed, the next patient was randomized to receive the same ropivacaine concentration (with a probability of 0.89) or 0.025% less (with a probability of 0.11). After any block failure, the concentration was always increased by 0.025% for the next. The study ended when 45 successful blocks were obtained.

We defined effective quadratus lumborum block as a resting visual analog score ≤ 3 and the absence of a need for rescue anesthetics.

**Results:**

The 90% minimum effective concentration of ropivacaine was 0.335% (95% CI 0.306 to 0.375%), and the 99% minimum effective concentration was 0.371% (95% CI 0.355 to 0.375%). The sufentanil consumption was 11 (11,13) and 24 (22,27) μg at 12 and 24 hours after quadratus lumborum block, respectively.

**Conclusions:**

The optimum dosage of ropivacaine is a 25 ml volume of 0.335% for quadratus lumborum block after cesarean delivery.

**Trial registration:**

The study was registered in the Chinese Clinical Trial Registry (No. ChiCTR2000040415).

**Supplementary Information:**

The online version contains supplementary material available at 10.1186/s12871-022-01954-5.

## Background

According to the US national report, approximately 1.3 million patients accepted cesarean section during 2018 [1]. It is noted that cesarean section is associated with severe pain after surgery. Krohg et al. reported that the pain intensity reaches > 6 after cesarean section [2]. In addition, severe pain disturbs mother-newborn bonding and breast feeding.

Quadratus lumborum block (QLB) was recently proposed as an alternative technique for post-cesarean delivery analgesia [3–6]. It involves local anesthetic injection into the thoracolumbar fascia adjacent to the quadratus lumborum muscle, which may facilitate the spread of local anesthetic into the thoracic paravertebral space [5]. QLB significantly reduces opioid requirements and improves analgesic effects after cesarean delivery [6, 7]. Several meta-analyses have proven that bilateral QLB significantly reduces 24-hour opioid consumption after cesarean section [7–9]. However, various concentrations of local anesthetics were used in previous studies; for example, 0.375% ropivacaine was administered in Hansen’s study [9], whereas 0.2% ropivacaine was used in Kang’s work [10]. There is not a definite optimum concentration of local anesthetics, which fluctuates from 0.125 to 0.375% [4, 11–13].

We speculate that an adequate concentration of local anesthetics could facilitate analgesic effects after cesarean delivery. Because QLB is usually administered in a single shot, the minimum effective concentration in 90% of patients (MEV90) might be more clinically valid than 50%. We applied a biased coin design (BCD) up-and-down method (UDM) (BCD-UDM) to explore the MEC90 of ropivacaine (Naropin®, AstraZeneca) in QLB for satisfactory analgesia post-cesarean delivery.

## Methods

This was a prospective, single-blind, dose-finding trial. The study was approved by the Clinical Trial Ethics Committee of Chengdu Women’s and Children’s Central Hospital (No. 2020119) and was registered in the Chinese Clinical Trial Registry (No. ChiCTR2000040415).

### Patient enrollment

After obtaining written informed consent, we prospectively enrolled patients undergoing selective cesarean delivery from December 2021 to March 2022 at Chengdu Women’s and Children’s Central Hospital. Inclusion criteria were age between 18 and 40 years, American Society of Anesthesiologists (ASA) physical status IorII, and body weight between 60 and 80 kg. Exclusion criteria were refusal to participate in the study, allergies to local anesthetics, preexisting neuropathy, coagulopathy, and postpartum hemorrhage. Eliminating criteria were block failure such that the sensory block level was behind T8, toxicity of local anesthetics, and no compression of the uterus within 2 hours after QLB.

### Ultrasound-guided QLB

The patient was routinely monitored in the operating room and PACU. The surgery was completed under combined spinal-epidural anesthesia. Ketorolac (30 mg) was injected intravenously at the end of the surgery, and then a patient control analgesia pump with 1 μg/ml sufentanil was connected to the patient. PCA 2 ml and 0.5 ml/h continuous infusion via v-line were provided (maximal volume within 1 hour 10 ml). Rescue analgesia consisted of 30 mg ketorolac iv injection (no more than 4 times within 24 hours).

An experienced anesthesiologist who was blinded to the concentration assignment until study completion performed the QLB block and administered the medication. In the PACU, approximately 1 hour after the end of the surgery, when the patient felt mild abdominal pain, posterior QLB (Type II) was performed bilaterally in the supine position (Supplementary file 1).

A broadband (2.5–5 MHz) convex transducer was used.

For the QLB, the transducer was placed at the level of the navel and moved cranially from the midclavicular to the midaxillary line with the 3 abdominal wall muscles clearly identified, and the aponeurosis of the external oblique muscle was visualized. Then, the quadratus lumborum muscle was found underneath the internal oblique muscle. An inner stylet of a 20 G intravenous catheter was advanced in-plane under US guidance in the anteroposterior direction through the muscle layers of the abdominal wall. The needle tip was at the thoracolumbar fascia between the quadratus lumborum muscle and the erectus muscle, which was typeIIof QLB block. Two milliliters of saline was injected to verify the needle position. On each side, a volume of 25 ml of the assigned solution was then injected under repeated aspiration.

The upper sensory block level was evaluated by testing the skin sensitivity to iced water in the midclavicular line bilaterally 1 hour after completion of QLB. The pain intensity was evaluated by the visual analog scale (VAS). Motor block was evaluated according to the Bromage scale (0 = full flexion of the feet and knees, 1 = just able to move the knees, 2 = able to move the feet only, and 3 = unable to move the feet or knees).

We defined effective QLB only if the patient reported a resting VAS score of ≤3 and had no requirement for rescue analgesics (i.e., PCA = 0), even after compression of the uterus within 2 hours after completion of the block. The block was considered ineffective if the patient needed additional analgesics using PCA or if the resting VAS score was more than 3 within 2 hours after the QLB.

### Biased coin design up-and-down sequential method (BCD-UDM)

The concentration assignment was carried out using a BCD, where the concentration of ropivacaine administered to each patient depended on the response of the previous patient. The first patient recruited received 25 ml of ropivacaine 0.25% based on clinical practice and a pilot study. If a successful block was observed with the first patient, the next patient was randomized to receive the same ropivacaine concentration (with a probability of 0.89) or to receive a concentration 0.025% less (with a probability of 0.11). In contrast, if any block insufficient was identified, the concentration was always increased by 0.025% for the next subject. The study ended when 45 successful blocks were obtained. A maximum concentration of 0.4% ropivacaine was set as the upper limit. This means that if a patient experienced a failed block when administered the maximal concentration, the concentration administered to the following patient would not be increased.

The 44 numbered, randomized assignments for successful blocks were generated by a computer and sealed in 44 envelopes by a resident. The envelope preparation and drug preparation were accomplished by a resident who took no further part in the study.

### Primary outcomes

These were the resting pain score and total volume of sufentanil consumption at 0, 1, 2, 4, 12, and 24 hours after QLB completion.

### Secondary outcomes

These were the pain score upon pressing of the abdomen and the bromage score at 0, 1, 2, 4, and 12 hours after QLB completion.

Patient characteristics and block complications within 24 h were recorded.

### Implementation and blinding

QLB performance and data recording were performed by Rong Cao and Linmei Deng, who were blinded to the interventions. Jing Yang enrolled participants and prepared injections according to the randomized allocation. Sequence generation and analysis were performed by Xuehan Li.

### Statistics

To estimate MEC90, a sample size of at least 20 (and at best, over 40) was recommended by Stylianou et al. after performing extensive trials [14, 15]. Therefore, we choose a minimum of 45 positive responses to accommodate potential dropout. We prospectively recruited patients until 45 positive responses were obtained.

Data are reported as the median (interquartile range) and mean (SD) as appropriate. Categorical variables are presented as numbers (proportions). Statistical analysis was carried out using the R statistical software package, version 3.2.1 (2015 The R Foundation for Statistical Computing, Vienna, Austria; ISBN 3–900,051–07-0, URL http://www.r-project.org) and SPSS 22 (SPSS Inc. USA).

The MEC90 was calculated using isotonic regression, and the 95% confidence interval (CI) was derived from the 2000 bootstrap replicates. Further analysis was performed using isotonic regression and bootstrapping CI to calculate the minimum effective concentration for a 99% success rate of block (MEC99) [16].

## Results

**Fig. 1:**
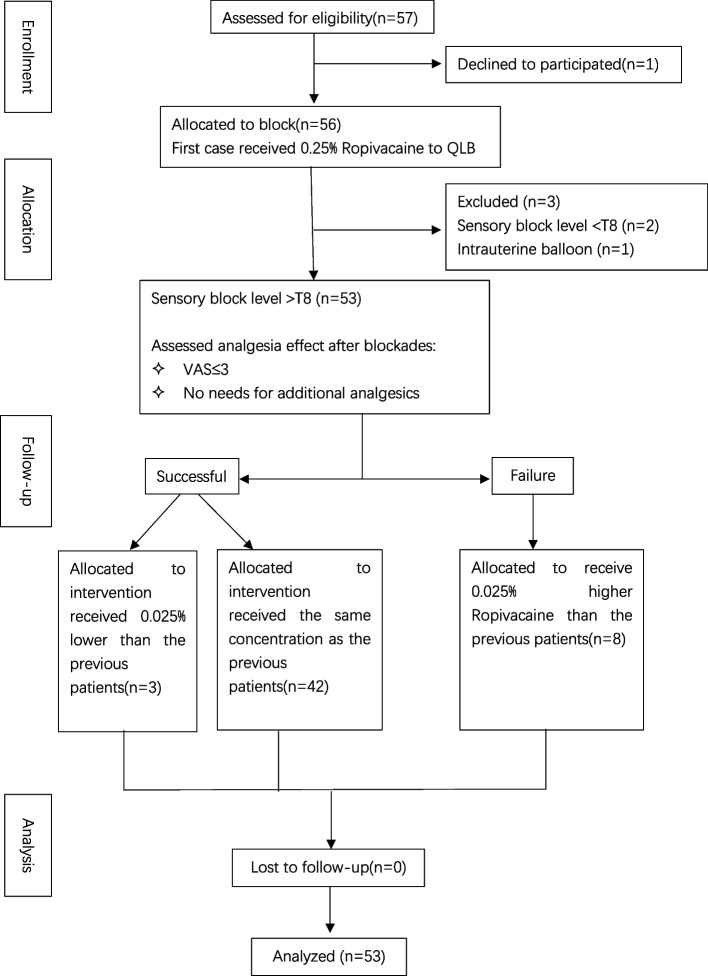
The study flow chart

A total of 56 patients were enrolled; 2 patients were excluded due to the sensory block level being lower than T8, and 1 patient was excluded due to the lack of abdominal pressing with an intrauterine balloon. 53 of the patients completed the study and were analyzed. The flow diagram for this study is shown in Fig. 1. The demographics and baseline characteristics are shown in Table 1. The median (IQR) age of the patients was 29 (27, 31). The duration of surgery was 41.0 (39.0, 48.0) min, and the interval time between the start of spinal anesthesia and the start of QLB was 140.0 (135.0, 150.0) min. Patient parameters comprising the pain score and sufentanil consumption are shown in Table 2.

**Table 1:** Demographic characteristics (*n* = 53)

Patient characteristics	Value
Age (years)	29(27, 31)
Weight (kg)	66.5(62.5, 72.0)
Height (cm)	159(157, 161)
Body mass index (kg/m2)	26.6(25.0, 27.7)
Duration of surgery (min)	41.0(39.0, 48.0)
Gestational age (weeks)	38.5 ± 1.7
Cesarean section history (%)	18 (34.0)
Interval time (min)	140.0(135.0, 150.0)
Levels	
T6	21 (39.6)
T7	24 (45.3)
T8	7 (13.2)
Comorbidities	
Anemia	3 (5.7)
Hypertension	5 (9.4)
Hypothyroidism	1 (1.9)
Diabetes	1 (1.9)
ICP	2 (3.8)

Note: Values mean are expressed as mean (SD), number (proportion) or median, (IQR) Intrahepatic cholestasis of pregnancy (ICP)

**Table 2:** clinic parameters

Time point (hours after QLB)	Pain score at rest	Pain score at pressing abdormen	Bormage score	Sufentanly consumption, mcg
0 h	2.0(1.0,3.0)	3.8 ± 2.3	2.0(1.0,3.0)	0 (0,0)
1 h	1.5 (1.0, 2.1)	4.34 ± 1.59	0 (0, 0)	1 (1, 1)
2 h	2 (1.5, 2.3)	4.4 ± 1.16	0 (0, 0)	2 (2, 4)
12 h	2.35 ± 0.68	4.415 ± 0.7012	0 (0, 0)	11(11, 13)
24 h	2.625 ± 0.6493	NA	NA	24(22, 27)

Note: Quadratus lumborum block (QLB)

**Fig. 2:**
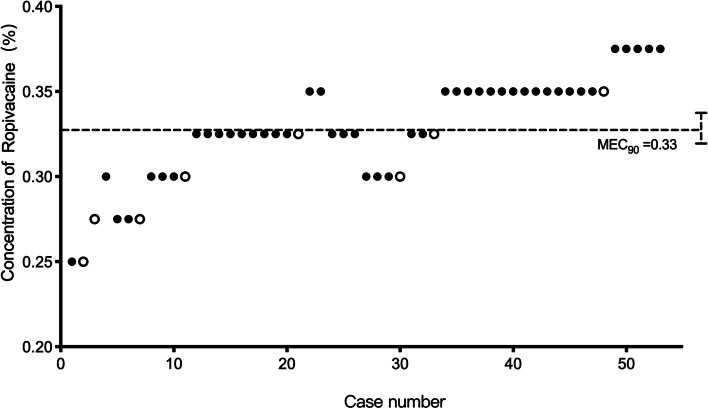
The biased coin design up-and-down sequence. Graph of successful (solid circle) and failed (hollow circle) quadratus lumborum block (QLB) with different ropivacaine concentrations. The horizontal line is the calculated minimum effective concentration of ropivacaine providing successful QLB in 90% of patients (MEC90)

The sequence of successful and unsuccessful QLB in patients recruited is presented in Fig. 2. The algorithm-adjusted response rates of the observed and pooled adjacent violators are presented in Table 3. The MEC90 of ropivacaine was 0.335% (95% CI 0.306 to 0.375%). By further analysis, the MEC99 was estimated to be 0.371% (95% CI 0.355 to 0.375%). No severe adverse events were detected during the QLB procedures, such as tinnitus, local anesthetic systemic toxicity, new-onset cardiac arrhythmia, nausea and vomiting, and hypotension.

**Table 3:** Observed and pooled-adjacent violators algorithm-adjusted response rates

Group Assigned concentration	Successful blocks, numbers	Trails, numbers	Observed response rate	PAVA-adjusted response rate
0.25	1	2	0.50	0.50
0.275	2	4	0.5	0.5
0.3	7	9	0.778	0.778
0.325	14	16	0.875	0.875
0.35	16	17	0.941	0.941
0.375	5	5	1	1

Note: Pooled-adjacent violators algorithm (PAVA)

## Discussion

Dixon’s up-and-down sequential method is a commonly used method to study the 50% minimum effective concentration (MEC50), but a 50% success rate cannot meet the needs of the clinical practice of a single shot. It has been considered imprecise [17] that the statistical methods applied to extrapolate the data to the relevant higher concentration of local anesthetics. Several recent reports have used the BCD method to determine the MEV90 or MEC90 of the local anesthetics used for regional blocks [16, 18]. This is considered a better method than Dixon’s up-and-down method. By utilizing the BCD-UDM, we found that the MEC90 of ropivacaine for QLB in parturients was 0.334 (0.3058, 0.3749), and that of MEC99 was 0.3708 (0.355, 0.375). In many countries, the officially recommended highest dose of ropivacaine is 225 mg [19], and bilateral 0.375% ropivacaine 25 ml for QLB is relatively safe.

The MEC90 of ropivacaine for a successful caudal epidural block in the female patient and a successful axillary brachial plexus block are 0.353 and 0.44%, respectively [17, 18]. Their definition of successful block is pain-free surgery without additional analgesics. In the present study, our definition was tolerable mild pain without the need for rescue opioids even after pressing the abdomen within at least 2 hours from T0. At that time, the effects of spinal anesthesia had worn off as the sensory block duration of ropivacaine in spinal anesthesia was 132.5 min [20]. It is reasonable that the concentration of ropivacaine for post-CD analgesia is lower than that for intraoperative analgesia. It was observed in our study that the resting pain VAS score might be less than 1 when the concentration is high, representing an adequate analgesic effect. Ropivacaine can preferentially block sensory nerves and has less effect on motor function [21, 22]. We observed that posterior QLB did not reduce leg movement (the bromage score was 0) when the concentration of ropivacaine was within 0.375%.

Generally, the effect of a single injection of QLB could spread to T7-L1 [23–25]. In the pilot study, we found that there was a higher rate of achieving T7 with 25 ml ropivacaine than with 20 ml. To ensure a sufficient level of sensory block, we advanced the needle along the paravertebral space after opening the fascia by injectate. Therefore, on the part of our patient, the sensory block level was T6, the same as for subcostal QLB [24]. The mechanism of this effect of QLB may be that the local anesthetic injected will not only spread in the fascial plane but also spread to the thoracic paravertebral space along the endothoracic fascia [5, 26]. Hence, QLB can block the somatic nerves and thoracic sympathetic trunk of the lower thoracic levels and abdominal viscera afferent nerves that share the same anatomical pathway of sympathetic nerves through the peripheral nervous system [27, 28]. Because of this, many studies have found that QLB has a better analgesia effect than TAP after major surgery [29, 30]. However, several anatomic studies have revealed the indeterminacy of the paravertebral spread [31–34]. A most recent net meta-analysis concluded that there is no significant difference between the analgesia effect of QLB and TAP [35]. This may explain the uncertainty of the pain score upon pressing of the abdomen in patients after CD. Post-CD pain can arise from incision, uterine contraction, and pressing of the abdomen to observe vaginal bleeding. Similar to the situation during the operation, when the abdomen is pressed, the visceral pain is not only of pelvic origin but also arises from other intra-abdominal structures, e.g., the peritoneum, which is innervated by sensory afferents reaching T5 [36]. Therefore, adequate analgesia for pressing pain requires a higher block level or other types of analgesia models.

Although QLB applied after CD is effective, its duration is not satisfactory enough in clinical practice. A higher concentration of local anesthetics can prolong the duration. Dam M et al. reported that the first opioid request was more than 10 hours of transmuscular quadratus lumborum block for percutaneous nephrolithotomy with 30 ml 0.75% ropivacaine [37]. In addition, 30 ml 0.375% ropivacaine was used for 5.2 hours after CD [9]. Another useful method is adjuvants such as dexmedetomidine. Dexmedetomidine used as an adjuvant has a longer time to first analgesic intake and necessitates less rescue analgesia in the erector spinae plane block [38].

Limitations: Because urinary catheters were pulled out 24 hours after surgery, we cannot exactly assess the first ambulation time. We did not assess the emotional state of the parturient, which might have influenced the patient’s need to receive analgesics and report pain scores.

## Conclusion

The optimum dosage of ropivacaine for QLB after CD is a 25 ml volume of 0.335% in patients of 60–80 kg body weight.

## Supplementary Information

**Additional file 1:** **Supplementary file 1.** Sonographic model image of transmuscular quadratus lumborum (TQL) block. The needle tip (red arrow) was at the thoracolumbar fascia between the quadratus lumborum muscle and the erectus muscle, which was typeIIof QLB block.

## Data Availability

The datasets used and/or analyzed during the current study available from the corresponding author on reasonable request.

## Abbreviations

QLB: Quadratus lumborum block
TAP: Transverse abdominal plane
CD: Cesarean delivery
MEC 90: The 90% minimum effective concentration
BCD-UDM: Biased coin design up-and-down sequential method
PCA: Patient controlled analgesia
VAS: Visual analog scale
PACU: Post anesthesia care unit
