# Supplementary figures and images for: The minimum effective concentration (MEC90) of ropivacaine for ultrasound-guided quadratus lumborum block for analgesia after cesarean delivery: a dose finding study
Source: BMC Anesthesiol. 2022 Dec 29;22:410. doi: 10.1186/s12871-022-01954-5 (PMC9798625; doi:10.1186/s12871-022-01954-5)

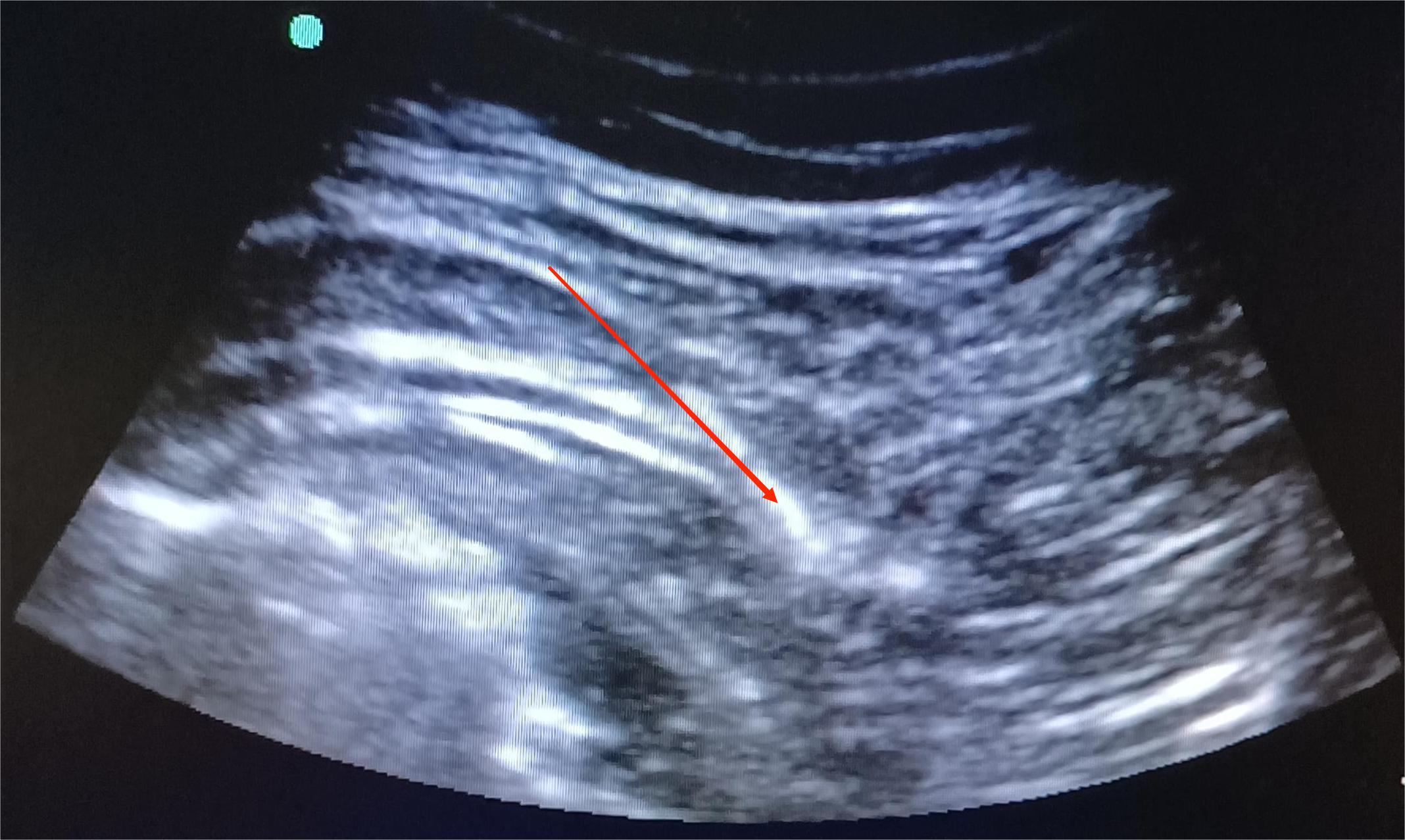

Supplement: Supplementary file 1 — Additional file 1: Supplementary file 1. Sonographic model image of transmuscular quadratus lumborum (TQL) block. The needle tip (red arrow) was at the thoracolumbar fascia between the quadratus lumborum muscle and the erectus muscle, which was typeIIof QLB block. [file 12871_2022_1954_MOESM1_ESM.docx]
